# Swarm of lightsail nanosatellites for Solar System exploration

**DOI:** 10.1038/s41598-023-46101-3

**Published:** 2023-11-09

**Authors:** Giovanni Santi, Alain J. Corso, Denis Garoli, Giuseppe Emanuele Lio, Marco Manente, Giulio Favaro, Marco Bazzan, Giampaolo Piotto, Nicola Andriolli, Lucanos Strambini, Daniele Pavarin, Leonardo Badia, Remo Proietti Zaccaria, Philip Lubin, Roberto Ragazzoni, Maria G. Pelizzo

**Affiliations:** 1 Centro di Ateneo di Studi e Attività Spaziali (CISAS), Università di Padova, via Venezia, 15, 35131 Padua, Italy; 2 Consiglio Nazionale delle Ricerche - Istituto di Fotonica e Nanotecnologie (CNR-IFN), via Trasea, 7, 35131 Padua, Italy; 3 Istituto Italiano di Tecnologia, Via Morego 30, 16163 Genova, Italy; 4 Dipartimento di Scenze e Metodi dell’Ingegneria, Università di Modena e Reggio Emilia, via Amendola 2, 42122 Reggio Emilia, Italy; 5 Dipartimento di Fisica e European Laboratory for Non-Linear Spectroscopy (LENS), Università di Firenze, Via Nello Carrara 1, Sesto Fiorentino, 50019 Florence, Italy; 6 Technology for Propulsion and Innovation S.p.A. (T4i), Via Emilia, 15, 35043 Monselice, Padua Italy; 7 Dipartimento di Fisica e Astronomia, Università di Padova, via Marzolo 8, 35131 Padua, Italy; 8 Consiglio Nazionale delle Ricerche, Istituto di Elettronica, Ingegneria dell’Informazione e delle Telecomunicazioni, via Gradenigo, 6B, 35131 Padua, Italy; 9 Dipartimento di Ingegneria Industriale, Università di Padova, via Gradenigo, 6A, 35131 Padua, Italy; 10 Dipartimento di Ingegneria dell’Informazione, Università di Padova, via Gradenigo, 6B, 35131 Padua, Italy; 11 Department of Physics, University of California - Santa Barbara, Santa Barbara, CA 93106 USA; 12 Istituto Nazionale di Astrofisica, Osservatorio Astronomico di Padova, Vicolo dell’Osservatorio, 5, 35122 Padua, Italy

**Keywords:** Space physics, Astronomy and planetary science, Optics and photonics

## Abstract

This paper presents a study for the realization of a space mission which employs nanosatellites driven by an external laser source impinging on an optimized lightsail, as a valuable technology to launch swarms of spacecrafts into the Solar System. Nanosatellites propelled by laser can be useful for heliosphere exploration and for planetary observation, if suitably equipped with sensors, or be adopted for the establishment of network systems when placed into specific orbits. By varying the *area-to-mass ratio* (i.e. the ratio between the sail area and the payload weight) and the laser power, it is possible to insert nanosatellites into different hyperbolic orbits with respect to Earth, thus reaching the target by means of controlled trajectories in a relatively short amount of time. A mission involving nanosatellites of the order of 1 kg of mass is envisioned, by describing all the on-board subsystems and satisfying all the requirements in terms of power and mass budget. Particular attention is paid to the telecommunication subsystem, which must offer all the necessary functionalities. To fabricate the lightsail, the thin films technology has been considered, by verifying the sail’s thermal stability during the thrust phase. Moreover, the problem of mechanical stability of the lightsail has been tackled, showing that the distance between the ligthsail structure and the payload plays a pivotal role. Some potential applications of the proposed technology are discussed, such as the mapping of the heliospheric environment.

## Introduction

The increase of human activities in space, accompanied by the desire of interplanetary travels for scientific and commercial purposes, requires access to small, inexpensive, and easy-to-launch satellites. In order to keep costs down, nanosatellites should be the preferred configuration, as they offer low masses. However, the most common propulsion technologies rely on a large amount of chemical propellant, which needs to be carried by the spacecraft, thus increasing the mass and launch costs. For these reasons, great attention has been raised to the concept of propellant-free satellites, mainly referring to the use of solar sails.

The idea of a spacecraft propelled by the pressure of solar radiation was conceived in mid-1950s. Since then, many theoretical studies have been focusing on the key technologies characterizing the solar sail propulsion: structure and materials of the sail, the deployment phase system, orbital dynamics, and attitude control systems^1,2^. However, very few lab-scale experimental tests have been carried out, with an even lower amount of missions actually launched into space^3–5^. These missions have been especially focused on the deployment phase of the sail and on the operation of the related subsystems, rather than on the use of the sail as a propulsion system. Indeed, solar sail technology still presents some open issues. The main ones relate to the limited power of the Sun and the directionality of the thrust, which requires sophisticated attitude control solutions.

In order to overcome the limitations of solar sails, laser-based propulsion systems have been proposed^6,7^. These can be eventually realized using ultra-high-power laser arrays, which offer both thrusts of remarkable magnitude and the ability to act on the direction of the thrust vector. Herein we shall refer to laser-driven sails as *lightsail* and to the thrusting lasing technology as *Direct Energy Laser Propulsion* (${\text{DELP}}$).

More in general, the laser-based technology can either be propellant-based^8^ or propellant-free. In the first case, the thrust is due to the impulse provided by a flow of particles ejected by the ablation produced by the laser on the sail^9–11^, whereas in the second case, the thrust is provided by the momentum exchange between the incident photons and the sail^12–16^. The main advantage of the propellant-free approach is that the energy source is completely separated from the spacecraft itself, with the important benefit of not requiring any propellant onboard. Another proposed technology concept based on laser thrust is the *Laser Electric Propulsion* (${\text{LEP}}$). In this latter case, a laser is employed to supply energy to a photovoltaic system, which in turn generates the necessary electricity to power an electric propulsion system^17^. Differently from ${\text{DELP}}$, ${\text{LEP}}$ requires each nanosatellite equipped with its own propulsion system, with increasing cost when a large number of satellites are launched.

Recently, ${\text{DELP}}$ has been proposed as a way to reach relativistic velocities necessary to cover deep-space distances in a short period of time, as foreseen by the ${\text{NASA}}$ *Starlight* program^18^ and the *Breakthrough Initiative*^19^. The goal of these projects is to demonstrate the feasibility of the first interstellar mission, by targeting other stellar systems such as Alpha Centauri^14–16,20,21^. A laser system placed on the ground is conceived as a facility^22^ for recurring launches, making its implementation cost-effective: the spacecrafts released by a mothership and parked in Earth orbit can be hit by the laser beam and accelerated into space. In particular, the use of laser arrays enables modularity and scalability, both necessary ingredients for the achievement of extremely high power, which in some studies is expected to be in the gigawatt range^14,16,23,24^. In this respect, studies on the phase control for the coherent combination of beams are in progress^25^. The use of a high-power laser as a propulsive source brings into play the radiation pressure exerted on the lightsail, with values of a few orders of magnitude greater than what is envisaged for solar sails. This important difference determines obvious mechanical and thermal effects, which require dedicated studies on the sail stability and composition^26–32^. In particular, thin film and multilayer sails, photonic crystals, gratings and metasurfaces have been recently introduced to improve the efficiency and the stability of lighsails^33–48^. Furthermore, it is important to quantify the damage that the interstellar medium can induce on the lightsail and the payload^49^.

Although many proposed mission scenarios (e.g. ${\text{NASA}}$ *Starlight*) foresee the use of low-mass spacecraft, it is still unclear how these will be able to host sensors and telecommunication systems fully capable of data communication and (possible) telemetry from deep space. In fact, the need to communicate over large distances requires the use of adequate antennas in terms of power and gain. In order to limit the power demand as much as possible, the use of very narrow lobes could be an option, although with the drawback of requiring an attitude control system.

**Figure 1:**
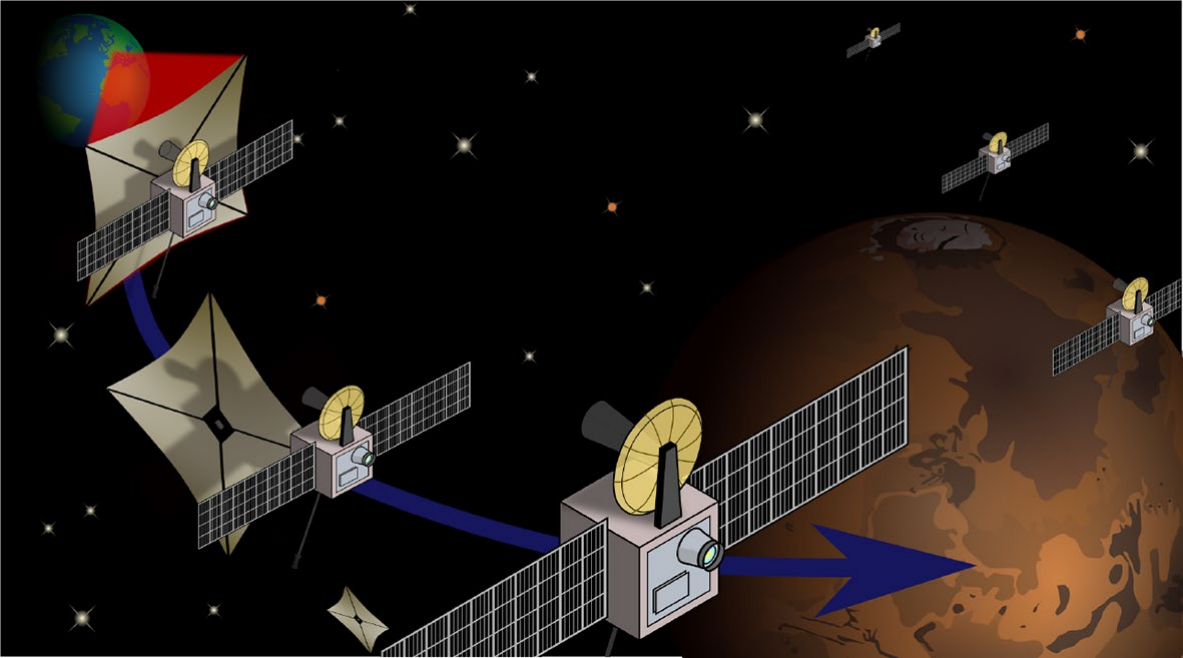
Concept of a swarm of nanosatellite propelled by laser.

The present work aims to overcome the limitations of the previously proposed approaches, enabling a new ${\text{DELP}}$-based mission, conceived to launch swarms of small satellites at non-relativistic speeds ($v \ll c$) to travel the Solar System, hence exploring the heliosphere and targeting planets (Fig. 1). ${\text{DELP}}$ will be assumed as the baseline technology for the development of Solar System missions, for example to Venus, Mars, and into deep heliosphere, In this regard, the power and telecommunication requirements for Solar System missions are less demanding than for deep space missions, even though many technological aspects remain challenging. Furthermore, by considering different mission targets, advantages and limits of the proposed technology will be discussed and compared to ${\text{LEP}}$.

The idea of exploring Mars by the use of a swarm of nanosatellites has been recently conceived^17,50,51^, including the case of propellant-based laser propulsion technology^51^. Vice versa, in the present work, we propose the first planetary mission based on nanosatellites propelled by ${\text{DELP}}$ by carefully analyzing all related technical aspects. Using ${\text{DELP}}$ technology, payloads of the order of 1 kg of mass can reach a planet like Mars or Venus in a few days. The non-relativistic speed reduces the demand in terms of laser power, making the mission more feasible. The weight of the payload is also pivotal to mechanically stabilize the complete nanosatellite system (lightsail plus payload), thus removing all the technological challenges related to stability posed by a few grams satellite for interstellar travel^52^. In Sect. "Propulsion and dynamics" the mathematics of propulsion and dynamics applied to the spacecraft is described, and the parameters for the orbital transfer to Mars and Venus are reported as an example. In Sect. "Payload and on-board systems" the payload and on-board systems of the mission are described. Besides careful considerations on the power system, the technology for the realization of the lightsail is also discussed in Sect. "Lightsail", assuming the thin film solution as the baseline^28,35^; in this respect, the mechanical and thermal stability of the lightsail during the thrust phase is analyzed. Finally, potential scientific applications of the mission are discussed in Sect. "Discussion and conclusion".

## Propulsion and dynamics

The key principle of propulsion in space is the need of expelling part of the mass carried by the spacecraft in order to produce thrust. Indeed, according to the Newtonian law of motion, the force *F* generated by a propulsion system has magnitude $\dot{m}V_\text{ext}$, being $\dot{m}$ and $V_\text{ext}$ the mass consumption rate and the effective exhaust velocity respectively, and has opposite direction in respect to the ejected particles. Space propulsion systems are then typically classified according to the physical processes used to eject the mass: chemical systems exploit a chemical reaction between solid and/or liquid propellants, while electric/magnetic systems accelerate ions through a tailored electric/magnetic field that mimics the behavior of a nozzle. Nevertheless, this concept describes a *classical* propulsion system. Indeed, when photons are the exhausted particles, the concept of mass consumption rate does not hold any longer. In a laser-driven propulsion system, the force $\overrightarrow{F_L}$ exerted on a flat non-diffusing lightsail can be written as^36^:

1 $$\begin{aligned} {\overrightarrow{F_\text{L}} }= \frac{I_0S}{c}\left[ 2R(\theta _i,\lambda _0)\cos {\theta _i}\hat{n} + A(\theta _i,\lambda _0)\hat{d}\right] \end{aligned}$$

where $I_0$ is the laser irradiance, *S* is the lightsail surface, *R* and *A* are the reflectance and absorbance of the lightsail computed at the angle of incidence $\theta _i$ and laser wavelength $\lambda _0$, $\hat{n}$ is the normal to the surface, $\hat{d}$ is the radial unit vector along the laser direction, and *c* is the speed of light. Eq. (1) provides the general expression, though for practical applications high reflectivity is required to maximize the force so that the absorption contribution can be neglected. Note also that, while in the case of the relativistic lightsails the Doppler-shift wavelength of the laser source has to be taken into account^36^, in the case of a lightsail used to drive an object at velocities $v \ll c$ the spectral performance can be optimized at the sole laser wavelength.

**Figure 2:**
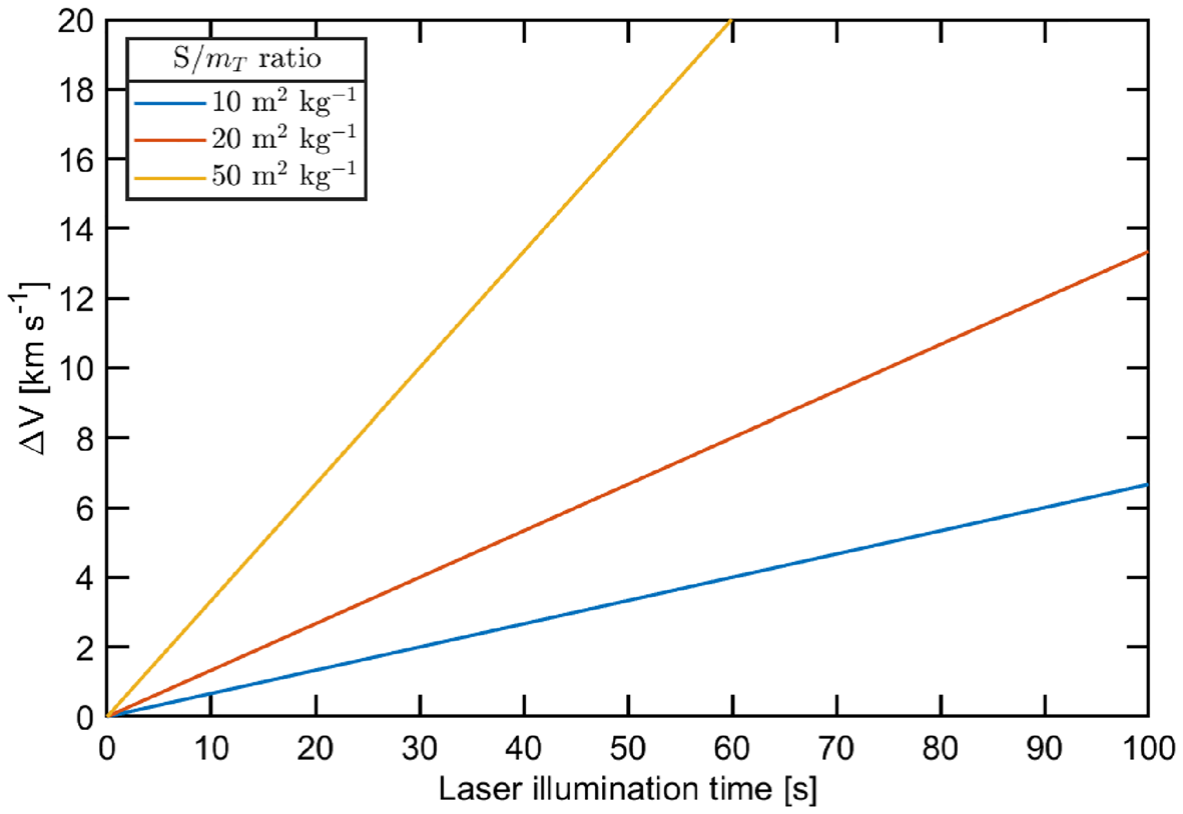
Dependence of the velocity gain modulus $\Delta V$ from the laser illumination time for different $S/m_\text{T}$ values. The simulation assumes $I_0=1$ GW m$^{-2}$ and departure from a geostationary orbit.

In the following discussion, it is assumed that a nanosatellite is accelerated from a circular parking orbit around Earth. In order to escape the gravitational attraction, the nanosatellite needs to increase its velocity by a *velocity gain* $\Delta \overrightarrow{V}$ and to be inserted into a hyperbolic trajectory arriving at the Earth Sphere of Influence (${\text{SOI}}$) with an *hyperbolic excess velocity* $v_\infty$ greater then zero. Under the assumption of a normal incidence reflection and considering a laser illumination time *t* short enough to consider constant the nanosatellite velocity vector, the $\Delta \overrightarrow{V}$ can be calculated from Eq. (1):

2 $$\begin{aligned} {\Delta \overrightarrow{V}}= \frac{\overrightarrow{F_\text{L}}}{m_\text{T}}t = 2\frac{I_0}{c}\frac{S}{m_\text{T}}t\hat{n} \end{aligned}$$

where $m_\text{T}$ is the spacecraft mass (i.e. the sum of the lightsail and nanosatellite masses). As shown in Fig. 2, when the laser is turned on at time $t=0$ s, $\Delta \overrightarrow{V}$ linearly increases with the laser illumination time following a slope depending on *S*, $m_\text{T}$, and $I_0$. Upon fixing the impinging laser irradiance $I_0$, the dependence on the *area-to-mass ratio* ($S/m_\text{T}$) has a direct impact on the performance of the accelerating spacecraft since it determines the time interval that the laser has to be activated to reach the desired modulus $\Delta V$. In turn, this activation time determines the costs associated with the launching phase. For instance, by assuming $I_0=1$ GW m$^{-2}$ and departure from a geostationary orbit, the time to achieve a $\Delta V$ of 5 km s$^{-1}$ is $\simeq 75$ s if $S/m_\text{T}=10$ m$^2$ kg$^{-1}$, but only $\simeq 15$ s if $S/m_\text{T}=50$ m$^2$ kg$^{-1}$. In general, a higher $S/m_\text{T}$ value is always to be preferred since it involves a higher thrust efficiency. Moreover, this value is a constraint on the spacecraft design as, for a given irradiance $I_0$, it defines the dimension *S* of the lightsail necessary to accelerate a mass $m_\text{T}$.

**Figure 3:**
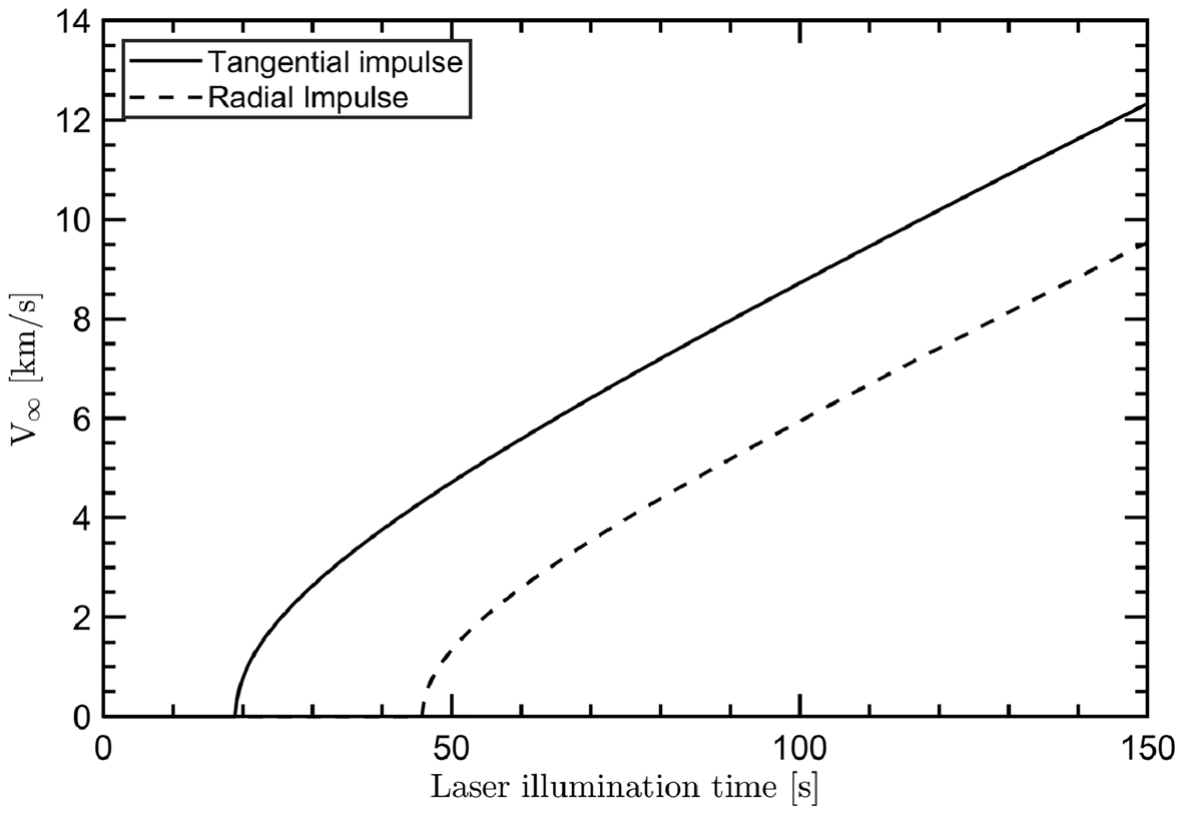
Hyperbolic excess velocity for a tangential and a radial impulse. The simulation assumes an area to mass ratio ($S/m_\text{T}$) equal to 10 m$^2$ kg$^{-1}$, $I_0=1$ GW m$^{-2}$ and departure from a geostationary orbit.

The location of the laser source is presently an open question for the scientific community. Many authors take the conservative assumption of having DELP placed on the Earth’s surface^17,35,36,51^, an approach typically referred to as ${\text{DELTA}}$ (Directed Energy Launch Technology Array). Nevertheless, an orbiting system named ${\text{DE}}$-${\text{STAR}}$ (Directed Energy System for Targeting of Asteroids and exploRation) has also been proposed^53^. This concept mainly derives from the need for a defense system against potential asteroid impacts, but it is intrinsically suitable also for realizing a laser propulsion solution. In the ${\text{DELTA}}$ scenario, the spacecraft experiences a radial ($\bot$) acceleration with respect to the orbit, while in the ${\text{DE}}$-${\text{STAR}}$ approach the spacecraft accelerates tangentially ($\Vert$). Hence, assuming that the laser thrusting phase is an impulsive maneuver, the hyperbolic excess velocity at the Earth ${\text{SOI}}$ can be expressed as:

3 $$\begin{aligned} \begin{aligned} (v^\Vert _\infty )^2&= {(v^\Vert )^2-2\frac{\mu }{r} =} \left( v_0 +\Delta V\right) ^2 -2\mu \left[ r_0^2+t^2\left( v_0+\frac{\Delta V}{2}\right) ^2 \right] ^{-1/2} \\ (v^\bot _\infty )^2&= {(v^\bot )^2-2\frac{\mu }{r} =} v_0^2 +\Delta V^2 -2\mu \left[ \left( r_0+\frac{\Delta Vt}{2}\right) ^2+(v_0t)^2 \right] ^{-1/2} \end{aligned} \end{aligned}$$

where $v^\Vert$ and $v^\bot$ are the speed at the end of the thrusting phase in the DE-STAR and DELTA scenario respectively, *r* is the spacecraft-Earth center distance at the end of the thrusting phase, $v_0=\sqrt{\mu /r_0}$ and $r_0$ are the velocity and the radius of the circular parking orbit, *t* is the laser illumination time, $\Delta V$ the velocity gain modulus as defined in Eq. (2) and $\mu$ is the standard gravitational parameter of the Earth. As shown in Fig. 3, a tangential impulse is generally more efficient than a radial one. Indeed, in the first case, the instantaneous velocity of the circular parking orbit and the acceleration direction are parallel to each other, and less energy is required to reach the target $\Delta V$.

**Figure 4:**
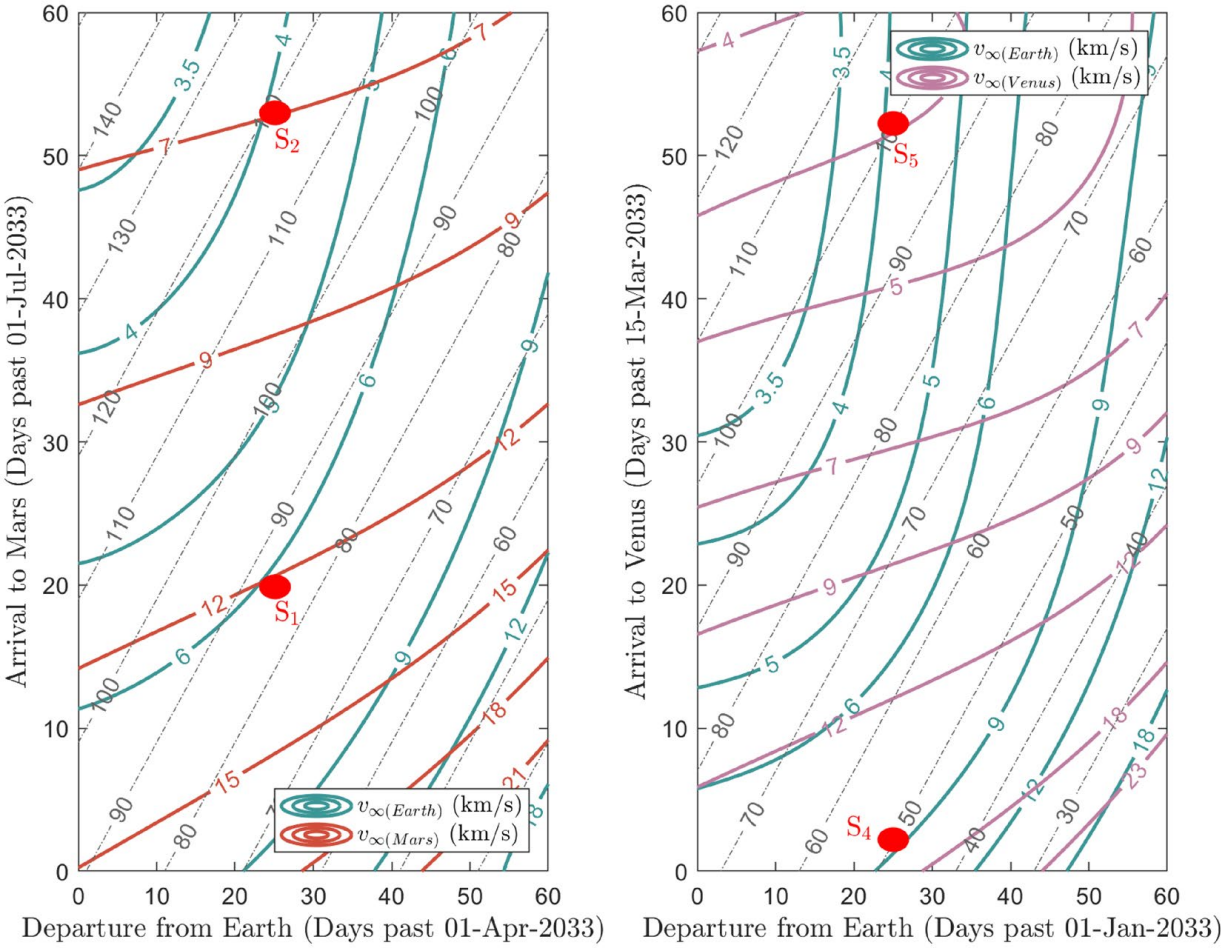
Porkchop plots of hyperbolic excess velocities v$_\infty$ in the case of Earth departure in a 2033 launch window for Mars (left) and Venus (right). The curves of ${\text{TOF}}$ reported are given in step of ten days and highlighted by the dashed grey lines.

In order to investigate the breakthrough capability of laser propulsion, the case of a spacecraft with an area to mass ratio $S/m_\text{T}$ of 10 m$^2$ kg$^{-1}$ travelling towards Mars and Venus propelled by a beam of irradiance of $I_0=1$ GW m$^{-2}$ is considered. Assuming a 2033 launch window, the astrodynamics solutions are found by solving the Lambert’s problem with a custom implementation of the algorithm provided in^54^ (more details are reported in the "Methods" section). Results are shown in Fig. 4, where the hyperbolic excess velocities at departure v$_\infty$(Earth) and arrival v$_\infty$(Mars/Venus) are plotted for a set of departure dates and times of flight (${\text{TOF}}$). In general, the ${\text{TOF}}$ obtained by using laser propulsion technology can be extremely reduced with respect to classical missions, namely not laser propulsion-based. For instance, simulations reveal that a journey to Mars with a departure on April 25${th}$ 2033 lasting 85 days would require a trusting phase of 69 s or 105 s for a tangential and a radial impulse respectively (solution labeled S$_{1}$ in Fig. 4 and Table 1). If the journey lasts 120 days, the lightsail is propelled for 43 s or 76 s (solution S$_{2}$ in Fig. 4 and Table 1), and if it lasts 200 days, the lightsail is propelled for 34 s or 66 s (solution S$_{3}$ in Table 1, not reported in Fig. 4 in order to avoid excessive stretching of the axes). Thus, the travel times are lower if compared with the ones obtained by assuming a simplify 2D Hohmann transfers, which are 259 days average in case of Mars. Similarly, to reach Venus by departing on January 25${th}$ 2033 in a ${\text{TOF}}$ of only 50 and 100 days, the lightsail needs to be propelled for 103 s or 141 s in the first case, and 43 or 76 s in the latter (see solutions S$_{4}$ and S$_{5}$ in Fig. 4 and Table 1).

**Figure 5:**
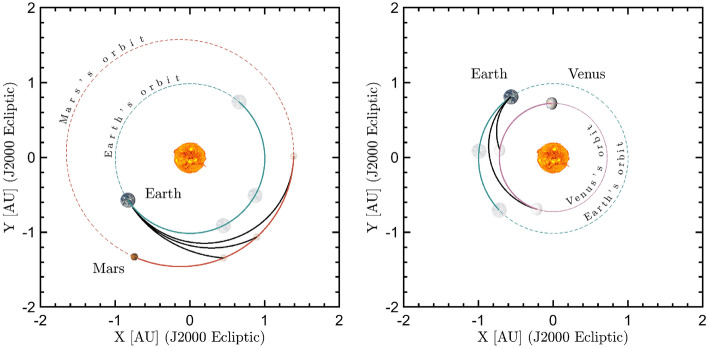
Orbital transfers to Mars (left) and Venus (right) for the parameters reported in Table 1. The planets at departure and arrival are shown in full and faded colors, respectively. The portion of the orbits covered by the planet while the spacecraft is travelling into the interplanetary medium are reported in solid lines, and the remaining portion in dashed line.

For comparison, it is worth to note that the average time of a Venus Hohmann transfer settles around 149 days. These results show also that the ligthsail need to be illuminated only for tens of seconds, so that the thruster time lasts only for a very limited time with respect to the journey; the lightsail can thus be released from the paylod after the propulsion phase, relieving also the mass of the nanosatellite. In Fig. 5 the simulation of spacecraft transfer orbits to Mars (left) and Venus (right) for the scenarios reported in Table 1 are shown. The simulations are performed by making a propagation of the position of the bodies (Earth, Mars/Venus and nanosatellite) in the gravitational field of the Sun using a custom implementation of the algorithms presented in^55^ (see "Methods" section). In^17^ ${\text{LEP}}$ is proposed as a potential technology capable to propel a cubesat in the Solar System, reaching Mars in a ${\text{TOF}}$ comparable with ${\text{DELP}}$. However, this technology is not very suitable for launching swarm of nanosatellites as each spacecraft needs to be equipped with its own proper motor, hence determining a mission cost increase. Instead, when applied to a single nanosatellite, ${\text{LEP}}$ requires a laser source carrying a power orders of magnitude lower than in the case of ${\text{DELP}}$ technology, which makes this technology competitive. This is due to the higher efficiency in converting the laser-transferred energy into thrust. However, there are few aspects that need to be carefully addressed. When ${\text{LEP}}$ propulsion is considered, a non-negligible percentage of the spacecraft mass (i.e. typically $> 60\%$ or more) is needed for the high-power electric motors and its fuel. Furthermore, in order to transfer all the energy necessary to obtain the desired $\Delta V$, the spacecraft has to be continuously illuminated by the laser for days (e.g. 5 days for a 4 kg payload and a total $\Delta V=5$ km s$^{-1}$), making the spacecraft pointing system a rather challenging task (during the illumination phase, the spacecraft moves of tens of thousands of km while Earth is rotating). Vice versa, in case of ${\text{DELP}}$, all the required thrust is given from the photons momentum transfer process, without the need of propellant or motors. Furthermore, by appropriately designing the laser array and sail sizes, the target $\Delta V$ can be achieved with a laser illumination of tens of seconds or, at most, of few minutes. During this time, the spacecraft typically moves of hundreds of km. This is certainly an aspect to take into account as it certainly simplifies the spacecraft pointing system. Reasoning in terms of total energy spent to keep the laser active and considering the mission scenarios reported in^17^, both technologies would spend hundreds of GJ kg$^{-1}$, with with a small saving in the case of ${\text{LEP}}$ with respect to ${\text{DELP}}$ when the target $\Delta V$ value is lower than $\simeq 15$ km s$^{-1}$. Finally, ${\text{DELP}}$ presents lower mission risks, as the loss of a satellite is less likely as the pointing system is less complex. Even assuming the loss of a satellite as a possible scenario, the cost of ${\text{DELP}}$-driven units is lower than for ${\text{LEP}}$-driven ones. Further discussion is needed to compare both technologies in case the mission profile foresees the launch of a single massive satellite towards a planet.

**Table 1:** Parameters for an orbital transfer from Earth to Mars and to Venus in a 2033 launch window.

Parameter	Units	Mars			Venus	
Solution label		S$_1$	S$_2$	S$_3$	S$_4$	S$_5$
Departure date		April 25 2033			January 25 2033	
Time of flight	days	85	120	200	50	100
v$_\infty$ at departure	km s$^{-1}$	6.30	4.01	3.12	8.90	4.03
Laser thrusting $\Vert$	s	69	43	34	103	43
$\Delta V_{\Vert }$	km s$^{-1}$	4.60	2.87	2.27	6.87	2.87
Laser thrusting $\bot$	s	105	76	66	141	76
$\Delta V_{\bot }$	km s$^{-1}$	7.00	5.07	4.40	9.41	5.07
v$_\infty$ at arrival	km s$^{-1}$	12.50	6.96	3.33	16.58	4.01

Another important aspect to consider is the velocity at arrival to be selected according to the mission profile, as it could result in a flyby, or in the insertion in the planetary orbit by means of proper maneuvers^51^. At the end of the transfer part, depending on the strategy adopted during approach, the spacecraft could be ballistically captured in orbits having dynamics which requires at least an high thrust maneuver to be stabilized and to reduce their eccentricity. Preliminary calculation on insertion maneuvers consider $v_\infty =0$ with respect to the target planet at the arrival. The velocity budget has been estimated using GMAT suite^56^) to be $\Delta v \simeq 900 - 1400$ m s$^{-1}$, depending on the desired final orbit eccentricity and altitude. A chemical thruster with about 3 N thrust would allow to perform a sufficiently fast maneuver. In this scenario, the mass of the nanosatellite is estimated to be increased by a wet mass of 5 kg; moreover, an increase of the mass of reaction wheels needs to be taken into account given the total mass increment.

## Payload and on-board systems

The perspective of launching a swarm of nanosatellites for heliosphere and planetary investigation with cost-effective laser propulsion requires a dramatic reduction of the payload mass and, in general, of the overall complexity of the spacecraft. The capability to send a high number of low cost satellites has the key advantage of redundancy, and the risk that some units experience a failure is largely compensated by the advantage of having multiple copies. When possible, commercially available *Components-Off-The-Shelf* (${\text{COTS}}$) have to be preferred since they have been proven to achieve high performance at increased functional density and low cost. However, they must be selected considering their radiation hardness, and, when necessary, tests should be carried out to prove their reliability^57^; in particular, semiconductor electronic components and integrated circuit devices (such as memories and microprocessors) should be tested in term of Total Ionization Dose (${\text{TID}}$) and Single Event Effects (${\text{SEEs}}$). The radiation hardening techniques involve logical (error-correcting code) and physical (shielding or redundancy) approaches; the latter one will increase the weight of the payload.

The selection and characteristics of subsystems are addressed according to the specific mission requirements, which is outside the scope of the present work. However, a first effort to identify the major subsystems of a payload for an interplanetary scientific mission is carried out^58,59^. First of all, the scientific nature of space exploration requires the adoption of a measuring system. As an example, here we refer to a specific sensor, being a planar Langmuir ion probe, which could be used to map the heliosphere plasma or to finely analyze a planet close environment. These type of sensors are very compact, as they consist of a fixed-bias flat-plate probe coupled with a dedicated electronics for the measurement of the collected ion current^60^. The weight is approximately 50 g for the envelope, electronics and sensor, while in terms of power the absorption peak is of 250 mW (Table 2).

A communication system able to acquire and manage the data provided by the sensor and to transmit them to Earth must be carefully selected. In particular, telecommunications at deep space distances (i.e. $>0.5$ au) require the use of a high gain antenna (${\text{HGA}}$) operating in either X-band or Ka-band^61^, with reflectarray (${\text{RA}}$) and mashed reflector (${\text{MR}}$) antennas being the only solutions in order to combine the ability to support any polarization, high efficiency, and low mass (i.e. an aerial mass density $\simeq 1-1.5$ kg m$^{-2}$)^62^. ${\text{RA}}$ antennas are characterized by low cost and very high reliability, but they are characterized by small bandwidths and typically support single-frequency links. On the contrary, MR antennas have large bandwidth allowing multiple-frequency operations, but have higher costs and are more prone to reliability issues. For instance, a MR antenna of only $D_\text{t}=30$ cm in diameter provides a gain $G_\text{t}$ higher than 25 dBi in X-band and 34 dBi in the Ka-band. Assuming a reasonable aerial mass density of 1.5 kg m$^{-2}$, the mass of this antenna can be estimated to be $\simeq 100$ g. Additional 100 g come from the mass of communication system electronics and cables, bringing the total weight of the telecommunications system to 200 g (Table 2). In order to evaluate a potential data transmission rate, the case of a satellite transmitting power of $P_0=$ 2 W and of a 70-m ${\text{NASA}}$ Deep Space Network (${\text{DSN}}$) receiver is considered. Such antenna gain $G_\text{r}$ is $\simeq 74$ dBi in the X-band and $\simeq 83$ dBi in the Ka-band, so that the estimation of the received signal power $P_\text{r}$ can be obtained by the Friis transmission equation:

4 $$\begin{aligned} P_\text{r}=P_\text{0}G_\text{t}G_\text{r}\left( \frac{c}{4 \pi f d} \right) ^2 \end{aligned}$$

where *f* is the carrier frequency and *d* is the satellite-receiver distance. In the mission scenarios discussed in this work, a distance $\simeq 2$ au need to be considered, so that the power of the received signal with a 70-m ${\text{DSN}}$ antenna is $\simeq -148$ dBm. The typical X-band noise temperature of such antenna is $T_\text{0}=25$ K^63^, which allows to achieve a Signal to Noise Ratio (${\text{SNR}}$) $>3$ dB in down-link. With such ${\text{SNR}}$ value, an appropriate choice of the modulation allows a down-link rate always higher than 3 kbps. The maximum antenna pointing error can be estimated by using the classical formula used for the diffraction from a circular aperture^64^. In particular, the $-3$ dB beam-width of a ${\text{MR}}$ antenna with circular shape is $\theta _\mathrm {-3dB}\simeq 70 \frac{c}{fD_\text{t}}=8.3^\circ$. If a maximum power loss of $L_\mathrm {\theta }=0.1$ dB is acceptable, the maximum tolerated pointing error $\theta _\text{P}$ is estimated to be

5 $$\begin{aligned} \theta _\text{P}\simeq \theta _\mathrm {-3dB}\sqrt{\frac{L_\mathrm {\theta }}{12}}=0.75^\circ \end{aligned}$$

To correct the attitude and pointing for communication purposes an *Attitude Determination and Control System* (${\text{ADCS}}$) can be used. It includes attitude sensors to determine the orientation, actuators to modify the attitude and reject disturbances, and a digital control link between the two. The vast majority of nanosatellites in orbit make use of simple magnetometers and Sun sensors to determine their pointing. Recently, high-performance micro-electromechanical systems (${\text{MEMS}}$) detectors and actuators have started to be used for high precision pointing, such as digital sensors, star trackers and gyros^65^; these new technologies offer the advantages of requiring limited volumes, weights and power consumption. The lack of a relevant magnetic field in the interplanetary space, and even in most planetary orbits, guides through the use of a reaction wheel-based attitude control systems in place of magnetic field actuators as magnetotorquer, with low-mass miniaturized reaction wheels already available in the market^66^. The commercial use for 1U cubesat, about the size of the proposed payload, assures that 3 micro reaction wheels with 3 mN s can be used, having a mass lower than 50 g per wheel. The use of reaction wheels requires momentum management in order to desaturate once the maximum speed is reached. The desaturation control actuator is normally obtained using magnetic torque coils, that are, as said, not applicable in lunar, martian or interplanetary orbit. Few alternative desaturation strategies for a reaction wheel precise pointing are available. In particular, the use of thrusters should be considered. For example, small water resistojet, which can achieve the necessary desaturation total impulse, have been recently developed for nano-satellite application in order to allow small maneuvers, such as collision avoidance and attitude corrections. Commercially available technologies have a very compact size (cube of 20 mm side) and weight of $\simeq 40$ g for each thruster, including propellant. The number of thrusters necessary to perform the desaturation task has been estimated to be 6, considering redundancy. The use of thrusters for attitude control without the reaction wheel is a possibility in case of loose pointing requirements. In particular, the pointing accuracy required by the telecommunication antenna does not appears particularly stringent and it could be satisfied even without reaction wheels.

The *Command and Data Handling* (${\mathrm {C \& DH}}$) system is responsible for supporting the ${\text{ADCS}}$ and the collection and communication of the data acquired by the sensors to the Earth. Heritage from previous missions will serve as a basis for the selection of high performance and reliable microprocessors to execute the flight software, to manage the subsystems, and to simultaneously interface with the sensors^67^.

Electrical power generation systems for nanosatellites have seen a continuous improvement from few watts in the past up to $10 - 20$ W available nowadays. Solar panels can be allocated along the sides of the spacecraft or can be deployed after the launch^65,68^. Currently, typical power densities for solar panels are in the $46-160$ W kg$^{-1}$ range at 1 au from the Sun^69^. By re-scaling such values for the Mars distance, where the solar irradiance is about 65% of that on Earth, the power densities to be considered are in the $30-104$ W kg$^{-1}$ range. Taking into account the mean power consumption estimated for all the satellite systems and summarized in Table 2, a conservative determination of the solar panels mass can be obtained by adopting the minimum value of power density (30 W kg$^{-1}$). As the total estimate of nanosatellite consumption is of about 3 W, most of which used by ${\text{ADCS}}$ and communication systems, the correspondent solar panel mass is calculated to be 100 g. In addition, a battery is required for the management of peak consumption (e.g. desaturation and communications) and to support the mission during eventual solar power interruption when attitude maneuvers or Sun eclipses are in place. The design of the battery capacity requires the definition of the energy that needs to be stored and the maximum tolerated depth of discharge (${\text{DoD}}$); reasonable values are two times the absorbed mean power for the capacity (i.e. $\simeq 6$ Wh) and a ${\text{DoD}}$ of 60%. For instance, by using Li-ions batteries, which show an energy density value of 150 Wh kg$^{-1}$ with a inherent efficiency $\eta _\text{bat}=0.95$^69,70^, the total mass required by the battery is 100 g.

Table 2 summarizes the weight and power estimates considering a list of technologies available on the market, mainly selected among ${\text{COTS}}$; however, when needed, the reliability and radiation hardness may require particular space certified components.

**Table 2:** Mass and power budget considering the mean power consumption.

Subsystem	Technology	Weight [g]	Power [mW]
Sensor	PLP	50	250
Communications	Transmitter	100	2000
	Receiver	100	
ADCS	Sun sensor (coarse)	40	100
	Sun sensor (fine)	5	40
	Reaction wheels (3)	150	300
	Desaturation thrusters	240	30
C &DH	Core PCB	5	–
	CPU	1	5
T/S	Alloy support	100	
	Filler/harness	20	
	Blanket	60	
Power	Solar array	100	(- 3000)*
	Battery	100	
	PPU	20	50
Total estimate		1097	2920

The power system is sized according to the estimated total and it is expressed as a negative number computed at the Mars distance *, not included in the total sum.

In the case of the ${\text{DELP}}$ technology, the lightsail needs also to be included in the mass budget. The weight of the lightsail depends on the membrane substrate, the nanostructure functionalizing it, and the boom-supporting structure. In the present work, the thin film technology is proposed for the lightsail realization^28^. Thin film of dielectrics are deposited on a substrate, which can be realized as a 10 $\mu$m thick Kapton membrane, or, even lighter, as a few hundreds of nm Silicon Nitride membrane. Many possible designs of boom structures exist, though the most used one has the main sail divided into four sections (or petals) and connected in a cross configuration. The boom can be realized in carbon-fiber-reinforced plastic^71^. The total mass $m_\text{l}$ of a lightsail comprising membrane substrate sustained by a *N*-petals boom supporting structure can be estimated by

6 $$\begin{aligned} m_\text{l} = S\rho _\text{s} t_\text{s} + \frac{N}{2} \alpha \sqrt{S}\cdot \tilde{\rho }_\text{b} \end{aligned}$$

where $\rho _\text{s}$ and $t_\text{s}$ are the density and thickness of the membrane substrate respectively, and $\tilde{\rho }_\text{b}$ is the mass per unit length of the booms expressed in kg m$^{-1}$. The parameter $\alpha$ depends on the shape of the lightsail: for a squared shape $\alpha =\sqrt{2}$, whereas for a round shape $\alpha =\sqrt{\frac{4}{\pi }}$. During the laser thrust phase, this weight needs to be taken into account for the estimation of the total mass, while it must not be accounted once the sail has been detached. For instance, in a worst-case scenario, a $S=10$ m$^2$ area sail realized with a Kapton membrane (i.e. $\rho _\text{s}=1420$ kg m$^{-3}$, $t_\text{s}=10$ $\mathrm {\mu }$m) and sustained with a four-petals carbon-fiber boom structure (i.e. $\tilde{\rho }_\text{b} =0.015$ kg m$^{-1}$^71^ in the present study) has a mass of 276 g if the shape is square or 249 g if the shape is round; the multilayer coating weight is negligible and not here considered. In the acceleration phase the laser beam induces on the lightsail a pressure that in a non-relativistic regime is given by^28^

7 $$\begin{aligned} P=\frac{I_0}{c}(A+2R) \end{aligned}$$

For instance, considering an ideal lightsail ($R=1$, $A=0$) and a laser irradiance $I_0=10$ GW m$^{-1}$, the pressure is $P\simeq 70$ Pa. This pressure must remain lower than the limit which induces a mechanical failure on the lighsail membrane. In this application, plastic deformation is considered unacceptable, and thus the lightsail material yield strength $\sigma$ is used to derive a maximum pressure value sustainable. Therefore, by considering the yield strength as the threshold at which material strain changes from elastic deformation to plastic deformation, and admitting a maximum deformation of the order of the lighsail diameter, the maximum pressure that a surface can withhold within elastic deformation can be estimated by^72^

8 $$\begin{aligned} P_{max} = \sigma \frac{t_s}{\alpha \sqrt{S}} \end{aligned}$$

**Figure 6:**
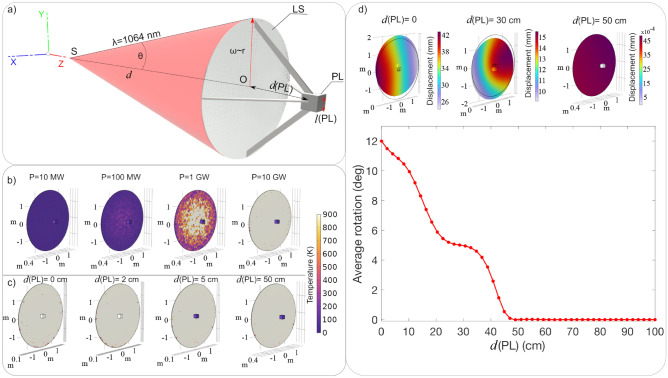
(**a**) Schematic representation (not in scale) of the nanosatellite, comprising the lightsail ($\text{LS}$) and the payload ($\text{PL}$). (**b**) Temperature analysis for different laser powers *P*, being 10 MW, 100 MW, 1 GW and 10 GW, in the case $d(\text{PL})=50$ cm. (**c**) Temperature analysis for $P=10$ GW varying the distance $d(\text{PL})$. (**d**) The maps (top panel) and the plot (bottom panel) report the deformations and the average rotation, respectively, occurring on the lightsail as function of the distance $d(\text{PL})$.

As an example, considering a 10 $\mu$m thick Kapton membrane, characterized by a $\sigma \simeq 6.9 \cdot 10^7$ Pa and area $S=10$ m$^2$, the maximum calculated pressure is $P_{max}\simeq 154$ Pa if the shape is square and $\simeq 193$ Pa if the shape is round. The analysis carried out is conservative and demonstrates that no mechanical resistance issues during the acceleration phase should emerge in a lightsail with a classical four-petals boom supporting structure if the laser beam has an intensity $< 20$ GW m$^{-2}$. For higher intensity values, a $N-$petals boom supporting structure with $N>4$ needs to be used to increase the mechanical strength. For example, considering a structure with eight petals, the total mass increases to 410 g (i.e. 48% higher than the four-petals case) if the shape is square, and to 356 g (i.e. 43% higher than the four-petals case) if the shape is round. An additional part to be considered is the mechanical structure connecting the payload to the lightsail. The structure can be envisioned as *M* booms, as reported in Fig. 6a. According to Table 1, such structure needs to be robust enough to effectively transfer the acceleration to the payload, which need to potentially sustain up to $\simeq 7g$. Under the assumption of uniform and on-axis laser illumination, the force required to impart an impulsive acceleration of 7*g* to the payload through the boom system is given by $F=m_P \cdot 7g$, with $m_P=m_T-m_L$ being the payload mass. Consequently, using some simple geometry, the force undergone by each boom can be calculated as

9 $$\begin{aligned} F_{b}=7 \frac{m_P \cdot g\sqrt{d(\text{PL})^2+\frac{\alpha ^2}{4}S}}{M \cdot d(\text{PL})} \end{aligned}$$

where $d(\text{PL})$ is the payload-lightsail distance, *S* is still the area of lightsail and $\alpha$ its the shape factor. Considering a system of four booms (i.e. $M=4$), a lightsail of area $S=10$ m$^{-2}$ and a payload-lightsail distance of $d(\text{PL})=0.5$ m (see next section), the force experimented by each boom is $F_{b}\simeq 80$ N for a square sail and $F_{b}\simeq 65$ N for a round sail. It is essential to size the the boom cross-section in order to provide sufficient strength, while simultaneously reduce the overall weight of the structure. By assuming hollow circular booms made in carbon fiber, having a diameter of 20 mm and a thickness of 1 mm, the calculated total compressive strength for each of them is $\simeq 2.5-3$ MPa, values which are fairly below the ultimate strength, which is in the GPa range. For this type of booms, the average density of the carbon-fiber is around 1.57 g cm$^{-3}$^73^, giving a total mass of 441 g for the four booms if the lightsail has a square shape, or of 357 g in case of a round shape. Thus, depending on the considered case, the system total mass (payload + lightsail + mechanical supports) is usually 1.5-1.8 times the payload mass $m_P$. In the case of ${\text{LEP}}$ technology, the satellite needs to include the electric motor and an adequate photovoltaic system, which increases the total mass by 2.5 times with respect to the payload mass^17^.

## Lightsail

A basic requirement is that nanosatellites are thermally and mechanically stable during the acceleration phase realized by means of the high power laser beam. Some recent papers discuss the thermal and mechanical stability of thin layer(s)^28^ and nanostructured lightsails^74^, but detailed analyses considering a configuration where the lightsail is part of a complete spacecraft comprising a small payload have not been reported so far. In the following discussion, a simple and efficient design is adopted for the ligthsail, consisting of a reflective thin film membrane (in particular TiO$_2$)^28^. Even though nanostructured lightsails offer the advantage that sophisticated surface patterns could be optimized to guarantee high heat dissipation and mechanical stability, a thin film design allows to reduce the overall complexity and manufacturing costs, still ensuring a good efficiency in terms of propulsion and thermal management^31^. Here, the previous analyses on thin film lightsails are extended considering a multiphysics approach (Finite Elements Methods - COMSOL Multiphysics - see "Methods" section) where different aspects are investigated at the same time, i.e. thermal and mechanical behaviours taking into account the expected optical response. A sail made by TiO$_2$, having a radius of 1.8 m (i.e. a total area of 10 m$^2$) and a thickness of 1 $\mu$m is considered for simulation. As demonstrated in^28^, the optical characteristics the TiO$_2$ thin film fully determine the thermal properties of the lightsail, which, in that case, was optimized for interstellar travel. In order to perform the present simulations, the optical constants used are those reported in^28^, and in particular the extinction coefficient *k* is assumed to be of $1\cdot 10^{-6}$. In addition, for the finite element method simulations, the following material parameters have been used: an expansion coefficient of $10.2\cdot 10^{-6}$ K$^{-1}$, Young’s module of 150 GPa, Poisson’s ratio equal to 0.26, and heat molar capacity equal to 58.2 J$\cdot$mol$^{-1}\cdot$K$^{-1}$. Jointed to the sail, a Si cube of $l(\text{PL})= 20$ cm side and 1 kg weight is considered as a representative payload (PL), as sketched in Fig. 6a. In the simulations, the lightsail and the payload are considered rigidly connected at distances $d(\text{PL})$ ranging from 0 up to 100 cm. A high-power laser of $\lambda _0$ = 1064 nm is used as source to push the sail. During the trusting phase, the sail should be ideally uniformly illuminated; however, here we consider a worse case scenario, in which the power distribution on the lightsail has a Gaussian profile, with a radius equal to the sail one. Indeed, the beam distribution at various distances will depend on the nature of the source and technology used to create a wide laser beam, but in the present simulations the illumination have been practically obtained using a very simple model, a single mode laser with divergence of 7.5$\times 10^{-8}$ rad and a lightsail at $d = 36.000$ km, which is the distance at which the thrusting starts. Different laser powers produce not only different pressure on the sail, but also different thermal effects, which can affect the operations and stability of the nanosatellite. In order to investigate this issue, different laser powers have been considered, being 10 MW, 100 MW, 1 GW and 10 GW. This last case corresponds to an irradiance of $I_0=1$ GW m$^{-2}$ on a 10 m$^2$ lightsail, which could be assimilated to the case discussed in Sect. Propulsion and dynamics. In Fig. 6b the thermal heating of the lightsail is reported as function of the laser power for the case d(PL)=50 cm. For powers of 10 MW and 100 MW, the lightsail has a temperature close to $T \simeq$ 50 K, while for a laser power of 1 GW the temperature reaches an average less than $T \simeq$ 600 K. Finally, for a laser power of 10 GW the lightsail is uniformly heated, reaching a value of $T \simeq$ 900 K, still sustainable by the material^28^.

The significant heating produced by the high power laser beam is not surprising and can have a not negligible impact on the payload. Therefore, simulations have been performed at different distances $d(\text{PL})$ in the case of $P=10$ GW. The results show that the silicon payload reaches temperature $T\simeq$ 900 K for $d(\text{PL})<5$ cm, as illustrated in Fig. 6c, while for $d(\text{PL})>50$ cm the payload can be considered thermally isolated from the sail. The thermal effects on the $\text{PL}$ can also induce displacement and rotations, hence instability, during the lightsail thrusting; hereafter, the term displacement indicates all deformations externally induced on the lightsail^74^. This physical quantity and related rotation effects are numerically simulated. The results show that for $d(\text{PL})$ up to 30 cm the entire systems has a non negligible displacement; on the contrary, for a $d(\text{PL})=50$ cm, the entire body of the sail moves without any perturbation (displacement $\sim 4\times 10^{-3}$ mm ) (Fig. 6d, top panel). It is thus interesting to evaluate the dependence of the rotation from the distance between the payload and the lightsail. Numerical study confirms that for a $d(\text{PL})>50$ cm the lightsail is not affected by any spin (Fig. 6d, bottom panel). As said, this analysis represents a worst case scenario, as the use of a larger laser beam waist/size is expected to reduce these effects.

**Figure 7:**
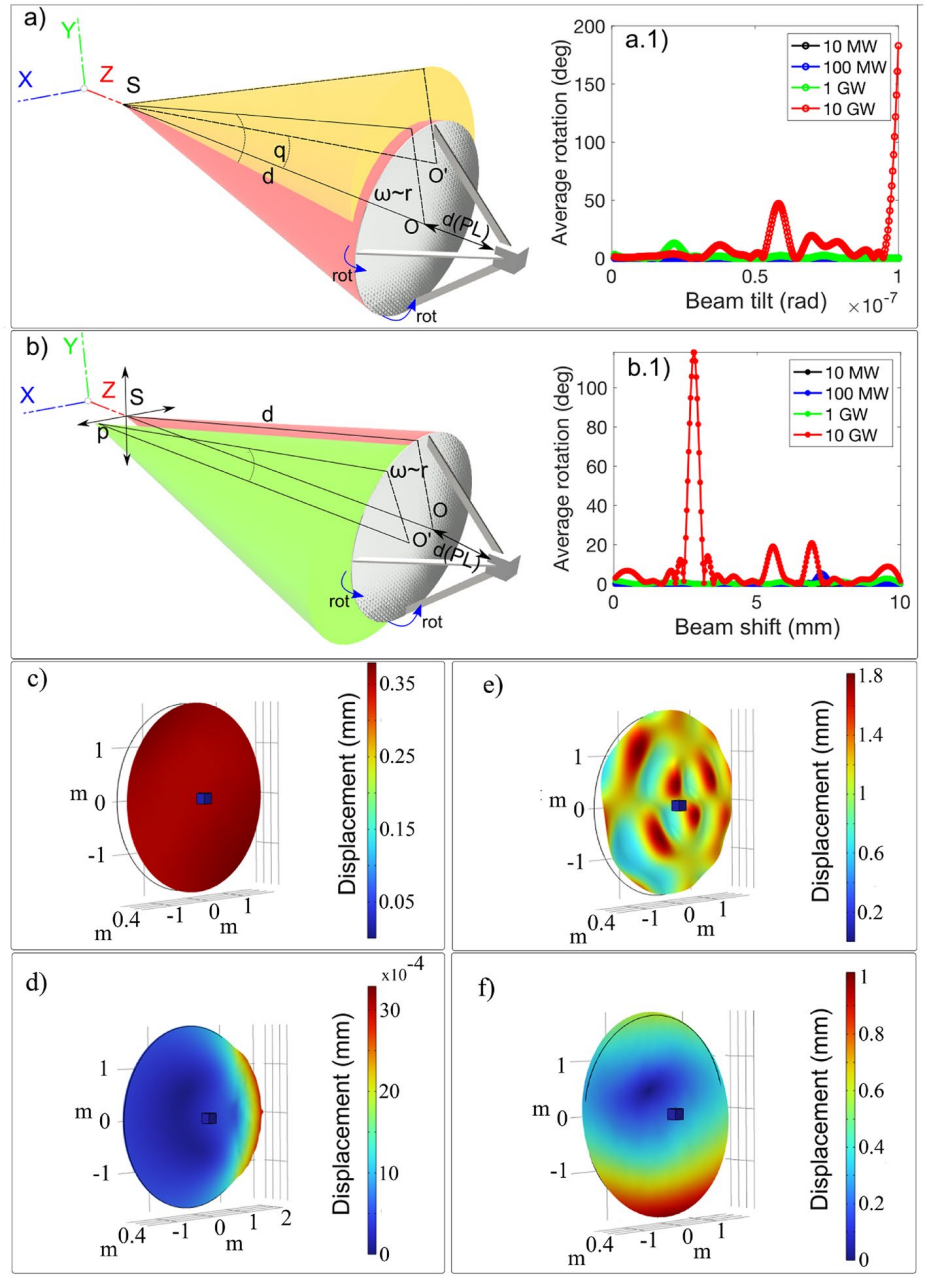
(**a**) Representative sketches (not in scale) of the beam tilt *q*, indicated in yellow. (**a**.**1**) Average rotation calculated by varying the laser power *P*, and *q* from 0.1$\times 10^{-7}$ up to 1$\times 10^{-7}$ rad. (**b**) Representative sketch (not in scale) of the source shift *p* illustrated in green. (**b**.**1**) Average rotation calculated by varying the laser power *P* and for *p* varying from 10 $\mu$m up to 10 mm. (**c**) Displacement map for beam tilt 1$\times 10^{-7}$rad. (**d**) Displacement map for $q=0.35\times 10^{-7}$ rad. (**e**) Displacement map for $p=0.035$ mm. (**f**) Displacement map for beam $p=3.2$ mm.

Another aspect to consider regards the misalignment between the beam Poynting vector and the lightsail normal. In detail, these numerical simulations are done considering a slightly beam tilt *q* with respect to the thrust axis and a source shift *p* in the plane perpendicular to the thrust axis, as depicted in Fig. 7a and b respectively. The lightsail average rotation for different laser beam powers and a beam tilt *q* ranging from 0.1$\times 10^{-7}$ up to 1$\times 10^{-7}$ rad is reported in Fig. 7a.1. These *q* values correspond to a beam shift at the lightsail surface ranging from $0.1\cdot D$ up to $1\cdot D$ respectively, where *D* is the lightsail diameter. In detail, the numerical study highlights that for $P=10$ GW and $q=1\times 10^{-7}$ rad the average rotation is 180$^\circ$, while for the same power but $q=0.35\times 10^{-7}$ rad no rotation occurs. Moreover, the lightsail dynamic obtained by varying the laser power and by applying a source (S) shift *p* from 10 $\mu$m to 10 mm has been evaluated (Fig. 7b) in order to study the stability at small perturbations regime. This corresponds to, for example, to potential vibrations which cause a relative shift between the lightsail and the laser source. For instance, with $P=10$ GW and $p=2.8$ mm the lightsail is subjected to a rotation of 120$^\circ$ (Fig. 7b.1). In addition, the displacement analysis highlights that varying the beam tilt and shift the lightsail is subjected to several deformations, as illustrated in Fig. 7c–f.

**Figure 8:**
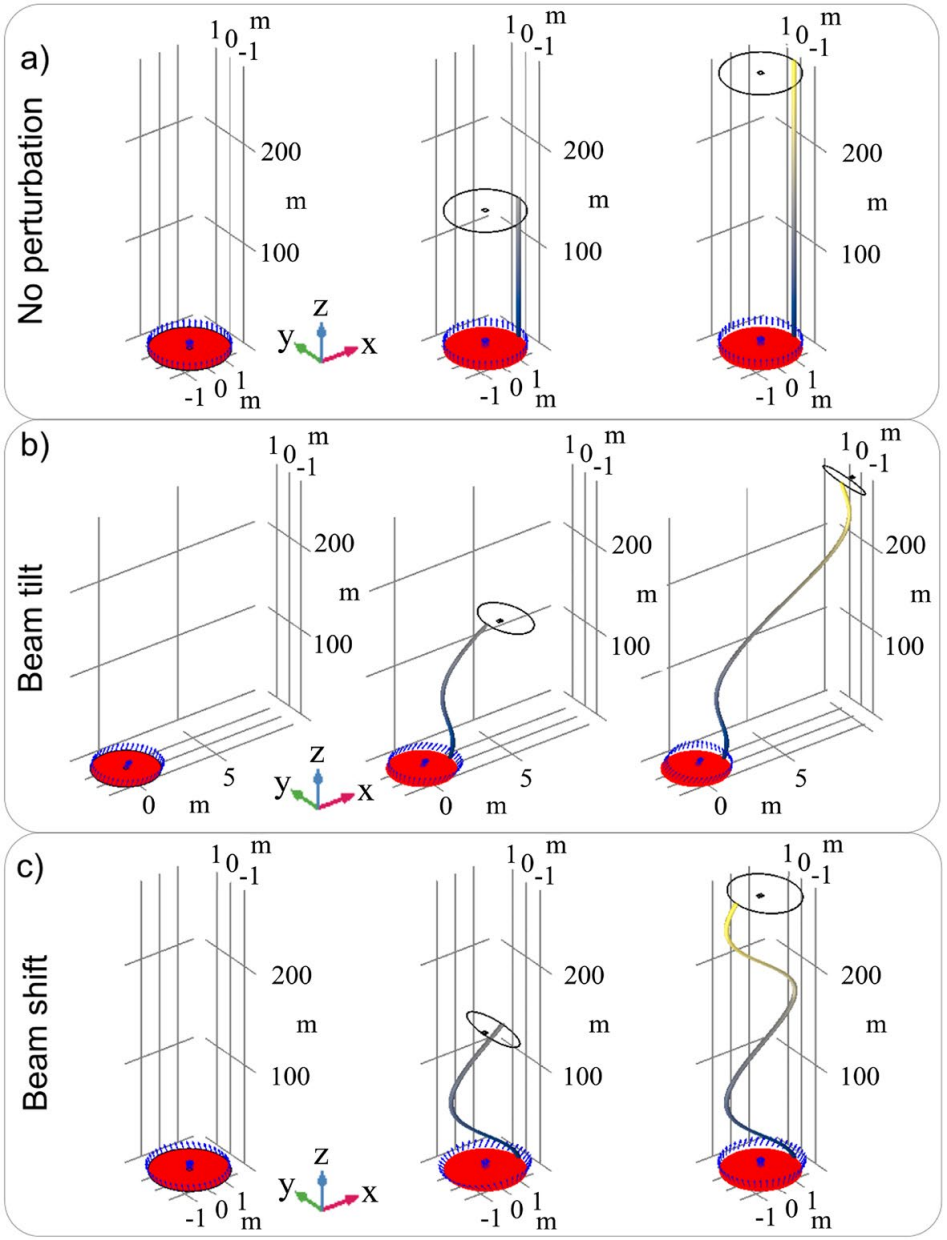
Numerical investigation of the lightsail trajectory; (**a**) the laser beam is nominal; (**b**) the laser beam is tilted of $q=1\times 10^{-7}$ rad; (**c**) the beam is shifted of $p=2.8$ mm. For each result, the initial lightsail position is indicated by the solid red object, while the black wireframe and blue arrows indicate the new position and the velocity direction along the new trajectory (gray-yellow tube gradient) respectively. For all the case studies $P=10$ GW. Each reported panel uses the following axis scale factor: 2 along *X* and *Y*, and 0.1 along *Z*.

The previous results led to a further interesting analysis, which shows how the lightsail trajectory changes consequentially to beam tilt *q* or shift *p*. In Fig. 8a, the simulation shows an unperturbed laser beam that impinges on the lightsail, and pushes it straight along its propagation direction *Z*. In the second case a laser beam tilted of $q=1\times 10^{-7}$ rad produces a spinning of the lightsail and a drift into a new trajectory (Fig. 8b). Finally, in the last case the beam is shifted of $p = 2.8$ mm, inducing a lightsail rotation along the propagation direction (Fig. 8c). These numerical studies highlight how a laser misalignment produces considerable effects, which can result in spacecraft drifting and spinning, even to the point of sail crumpling. Other issues could be related to atmospheric turbulence, which can play a major role in distorting the beam profile and thus the lightsail response. For the sake of simplicity, this aspect will be disregarded here and will be considered in a more detailed study in the future. Overall, simulations confirm that both for small and big perturbations, the system seems to be stable during the acceleration phase if the laser intensity is lower than 1 GW m$^{-2}$. In contrast, with a laser having intensity at 10 GW m$^{-2}$, some conditions of instability can arise both in small perturbations regime and big perturbations regime. Simulations demonstrate how complex is the realization and control of the illumination of the lightsail-payload system. Once the technology and design of the lightsail as well as the characteristics of the laser beam have been defined, it will be necessary to deeply study and simulate the various effects that can be induced by the radiation pressure, especially in terms of mechanical stability. For instance, using Eq. (1) it is possible to derive the force needed to push the lightsail at the speed of 10 km/s. Accordingly, it is possible to calculate the torque that affects the lightsail in the two worst cases analyzed before. For a beam tilt of 1$\times 10^{-7}$ rad the lighsail is subjected to a torque of 228 N$\cdot$m, while for a beam shift of 2.8 mm the values is if 84 N$\cdot$m. Hence, in both cases, the torque is too high to be compensated by suitable engines. For this reason, a lot of effort has been put in finding alternative solutions to stabilize the lightsail. Two classes of possible solutions have been analyzed: shaped sails, which are capable to re-orient themself without active feedback^33,42,52^, and plane nanophotonics structures and metasurfaces^38,40,41,44,47,75–77^, which take advantage of the electromagnetic field to contrast the torque applied. While these solutions have been proven to be effective for small lightsail areas, the scalability of such technologies over large surfaces needs to be demonstrated. Another crucial aspect to address is the precise tracking of the lightsail during the whole acceleration phase. For the DE-STAR configuration, the laser is placed in orbit as the lightsail; therefore, it is possible to adopt configurations in which the distances between the laser system and the lightsail are minimized to non-critical values, approximately tens kilometers, which makes feasible the tracking of the sail throughout the whole propulsion phase. In contrast, a distinct set of considerations arises for the DELTA configuration, wherein the laser is placed on the Earth’s surface, at hundreds or even thousands of kilometers from the lightsail. In principle, in this case, the laser would need an accurate lightsail tracking system to allow pointing correction required by any deviation of the orbit with respect to the predicted one, taking into account also the travel time of the laser beam. Undoubtedly, this would be one the most pivotal aspects of this propulsion scenario, which would require tracking systems with sub-arcsecond precision. However, it should be noted that in the DELTA configuration, the acceleration provided by the laser can be considered completely radial, effectively preserving the tangential velocity component except for small fluctuations due to misalignments, as already considered, and gravitational perturbations. Given this scenario, the complexity of the problem is relaxed. In conclusion, a detailed analysis of the effects of beam misalignment over lighsail, stability countermeasure, and potential adoption of a tracking system should carried out as an integrated project, which requires a system engineering approach.

## Discussion and conclusion

The theoretical scenario of reaching relativistic velocities makes ${\text{DELP}}$ technology extremely interesting for the exploration of deep space. However, tremendous technical challenges exist. In particular, the fabrication of a large area, extremely thin and stable lightsail appears out of reach due to current technology limitations. Furthermore, no clear strategies for slowing down a relativistic flying object exist, a crucial aspect when the object mission is performing observations or analyses^78,79^. Last but not least, the relativistic object needs to be equipped with a communication system of adequate size, mass, and power to send data back to Earth. Therefore, seeking a feasible mission scenario, in this article we discuss how ${\text{DELP}}$ can be applied for the exploration of the Solar System. In particular, ${\text{DELP}}$ technology is proposed for propelling swarms of nanosatellites into the Solar System, for example, to map different areas of the heliosphere or a specific magnetosphere. The effort of building a launch facility is justified by the multitude of nanosatellites that can be launched in sequence. With respect to deep space exploration, Solar System missions foresee relaxed requirements, even though they can be exploited for the development and test of key technologies, thus laying the groundwork for enabling the technologies for deep space exploration. In the present discussion, the ${\text{DE}}$-${\text{STAR}}$ and ${\text{DELTA}}$ laser facility concepts have been exploited, where the laser sources are positioned either in orbit or on Earth surface, respectively. However, a third, groundbreaking perspective is to place the laser system on the Moon’s surface, having great benefits such as the lower gravitational attraction with respect to the Earth, lack of atmosphere, no obstacles during the lighting phase and less risk of accidents. Although this solution is on a long-term time scale, it is however very well in line with the desire to create a human colony on the Moon^80^. Prior to the launch and in case of orbital release of nanosatellites, a first phase of orbit insertion should be planned by exploiting commercially available deploying platforms which can be mounted on a large variety of launch vehicles and can be preconfigured to accommodate any kind of satellite from 1U up to assemblies of 1$\times$12U, 2$\times$6U and 4$\times$3U^57^. A sail deployment phase should also be envisioned according to previous studies^81–84^. Considering that the sail will be used to reach the targeted velocity within a few hundred seconds and that no additional role for the sail is expected in the proposed mission, it could be reasonable to consider detaching the sail from the payload after the acceleration phase. This would enable an easier operation of the nanosatellites during the journey and at the target. In any case, our preliminary simulations show that the first thrusting phase is very complex and delicate and that the modeling of all the details of the illumination system is fundamental to guarantee a successful departure and acceleration. In particular, once the illumination system technology is defined, and the laser beam properties given, a detailed analysis of the mechanical stability of the system versus potential beam misalignment needs to be carried out. On the contrary, the thermal stability does not pose any concerns.

The designs and simulations reported in this work show that the proposed technology can be a valid option for developing a family of small satellites, capable of operating also as a cooperative network, for large-scale observations or for the mapping of the heliospheric environment. Indeed, in this last mission scenario, the nanosatellites would be radially propelled without the need for further orbital maneuvers. To date, the interplanetary environment, and in particular the heliospheric plasma, is only partially known due to the few existing opportunities for carrying out in-situ measurements, basically linked to scientific exploration missions^85^. The composition and characteristics of the heliospheric plasma remain defined mainly through theoretical models only partially verified. Therefore, there is an urgent need to perform a more detailed mapping of the heliospheric environment especially due to the growth of human activities in space. Consequently, the potential use of a large set (swarm) and low-cost nanosatellites equipped with optimized instrumentation can significantly improve the possibility of heliosphere characterization. Further exploitation can be the targeting of a planet such as Mars and to perform not just a flyby, but a capture. In this scenario, a swarm of nanosatellites can host different sensors, or it can be used to constitute a communication system network for a Martian colony. Finally, by increasing either the $S/m_\text{T}$ ratio or the laser illumination time *t* of a factor 5, values for $\Delta V$ much higher than $20 - 25$ km s$^{-1}$ can be obtained, enabling the possibility of reaching the helio-pause and even beyond in just a few years (i.e. heliocentric distances of $\simeq 100 - 130$ au in less than 10 years). Although within these last mission scenarios, there is the need to upgrade the communication system by adopting nuclear-type energy sources, resulting in higher mass budgets, higher costs, and more restrictive safety protocols, the possibility of launching swarms of nanosatellites to explore the most remote part of the heliosphere would still remain attractive and an exciting frontier to reach.

## Methods

### Orbit simulations

The astrodynamics solutions for Mars’ and Venus’ transfer orbits are computed by solving the Lambert’s problem to determine the fastest trajectories with lower cost requirements in terms of departure and arrival velocities. The mission scenarios selected are computed for the year 2033 when both a perihelic opposition of Mars and an inferior conjunction of Venus occur; however, this choice is not binding and, in general, it is possible to envision equivalent mission scenarios in other years. The departure windows considered start on April 1${st}$ 2033 for Mars and January 1${st}$ 2033 for Venus; then, for the 60 following days, the transfer orbit solution is computed, obtaining the required $\Delta V$, which is directly linked to the laser illumination time by using Eq. (2), the hyperbolic excess velocity at departure and at arrival, as well as the ${\text{TOF}}$ associated. The sets of solutions, obtained with a custom implementation of the algorithm provided in^54^, are shown in the porkchop plots of Fig. 4. For the selected solutions, the spacecraft and the planets’ trajectories are then simulated by numerically solving the differential equations of a 3D 4-body problem comprising the Sun-Earth-Mars/Venus-spacecraft system, the latter being massless. The numerical integration, performed at a step of 1 s, is based on the algorithm presented in^55^, which makes use of the *ODE45* solver in ${\text{MATLAB}}$. The evolution of the planets and spacecraft’s positions are computed in the *J2000* reference frame, while the final orbits are projected in the *J2000 ecliptic* reference frame for better graphical visualization (Fig. 5). The ephemeris of the planets at the departure time are computed by making use of the ${\text{MATLAB}}$ Interface to the ${\text{SPICE}}$ toolkit^86^ at the ${\text{DE440}}$ integration epoch.

### System mechanical and thermal stability simulations

The COMSOL-based model uses the ”Geometrical Optics” interface to trace the paths of rays through the lightsail system. A Gaussian beam source is used and it is placed 50000 km away from the target. The ”Heat Transfer in Solids” and ”Solid Mechanics” interfaces are used to model the thermal expansion and the displacements/ deformations and rotations of the proposed system. In order to consider the rigid movement of the entire system lightsail and payload in the mechanics has been used a prescribed velocity of 10.000 km h$^{-1}$, along Z, and the mass center has been calculated and included in the numerical simulation. In order to determine any rotation that can affect the lightsail the ”average rotation” node is added to the model. This node allows computing average rotation for a set of points (along the edges of the lightsail) with respect to the nanosatellite center of rotation. The ray trajectories and temperature distribution affect each other through a bidirectional coupling. In other words, the ray trajectories affect the temperature field, which in turn perturbs the ray trajectories, both directly and through the resulting structural deformation. To solve for the ray trajectories and temperature in a self-consistent manner, the dedicated Ray Heating interface and Bidirectionally Coupled Ray Tracing study step are used. The Bidirectionally Coupled Ray Tracing study step sets up a solver loop in which the ray trajectories and temperature are computed in alternating steps for a fixed number of iterations.

## Data Availability

Source data for figures are referenced within the paper; simulated data are available upon request to the corresponding author.

## Code availability

Code developed to make the astrodynamics simulations is available upon request to the corresponding author.

## References

[1] Gong S, Macdonald M (2019). Review on solar sail technology. Astrodynamics.

[2] Spröwitz, T. et al. Membrane deployment technology development at DLR for solar sails and large-scale photovoltaics. In 2019 IEEE Aerospace Conference, 1–20 (2019).

[3] Whorton, M., Heaton, A., Pinson, R., Laue, G. & Adams, C. L. Nanosail-D: The first flight demonstration of solar sails for nanosatellites. In 22nd Annual AIAA/USU Conference on Small Satellites (2008).

[4] http://sail.planetary.org/ Last access. 2019. http://sail.planetary.org/.

[5] Mori O (2010). First solar power sail demonstration by IKAROS. Trans. Jpn. Soc. Aeronaut. Space Sci. Aerosp. Technol. Jpn..

[6] Rezunkov YA (2021). High Power Laser Propulsion.

[7] Lubin P (2022). The Path to Transformational Space Exploration: Applications of Directed Energy.

[8] Levchenko I, Bazaka K, Mazouffre S, Xu S (2018). Prospects and physical mechanisms for photonic space propulsion. Nat. Photonics.

[9] Felicetti L, Santoni F (2013). Nanosatellite swarm missions in low Earth orbit using laser propulsion. Aerosp. Sci. Technol..

[10] Zhang T (2015). Macroscopic and direct light propulsion of bulk graphene material. Nat. Photonics.

[11] Phipps C (2010). Laser-ablation propulsion. J. Propuls. Power.

[12] Marx G (1966). Interstellar vehicle propelled by terrestrial laser beam. Nature.

[13] Forward RL (1984). Roundtrip interstellar travel using laser-pushed lightsails. J. Spacecr. Rockets.

[14] Lubin P (2016). A roadmap to interstellar flight. JBIS J. Br. Interplanet. Soc..

[15] Kulkarni N, Lubin P, Zhang Q (2018). Relativistic spacecraft propelled by directed energy. Astron. J..

[16] Lubin P, Hettel W (2020). The path to interstellar flight. Acta Futura.

[17] Sheerin TF, Petro E, Winters K, Lozano P, Lubin P (2021). Fast Solar System transportation with electric propulsion powered by directed energy. Acta Astronaut..

[18] https://www.deepspace.ucsb.edu/projects/starlight Last access. https : / / www.deepspace.ucsb.edu/projects/starlight (2021).

[19] https://breakthroughinitiatives.org/initiative/3 Last access. https://breakthroughinitiatives. org/initiative/3 (2021).

[20] Perakis N, Schrenk LE, Gutsmiedl J, Koop A, Losekamm MJ (2016). Project dragonfly: A feasibility study of interstellar travel using laser-powered light sail propulsion. Acta Astronaut..

[21] Kudyshev ZA, Kildishev AV, Shalaev VM, Boltasseva A (2021). Optimizing startshot lightsail design: A generative network-based approach. ACS Photonics.

[22] https://www.hilase.cz/en/success-stories/space-propulsion-and-space-debris-removalby- lasers Last access. https://www.hilase.cz/en/success-stories/ space-propulsion-and-space-debris-removal-by-lasers (2021).

[23] Parkin KL (2018). The breakthrough starshot system model. Acta Astronaut..

[24] Worden SP, Green WA, Schalkwyk J, Parkin K, Fugate RQ (2021). Progress on the starshot laser propulsion system. Appl. Opt..

[25] Bandutunga CP, Sibley PG, Ireland MJ, Ward RL (2021). Photonic solution to phase sensing and control for light-based interstellar propulsion. JOSA B.

[26] Davoyan AR, Munday JN, Tabiryan N, Swartzlander GA, Johnson L (2021). Photonic materials for interstellar solar sailing. Optica.

[27] Campbell MF, Brewer J, Jariwala D, Raman AP, Bargatin I (2022). Relativistic light sails need to billow. en. Nano Lett..

[28] Santi G (2022). Multilayers for directed energy accelerated lightsails. Commun. Mater..

[29] Rafat MZ (2022). Self-stabilization of light sails by damped internal degrees of freedom. Phys. Rev. Appl..

[30] Savu, D.-C. & Higgins, A. J. Structural stability of a lightsail for laser-driven interstellar flight. Acta Astronautica. https://www.sciencedirect.com/science/article/pii/S0094576522004684 (2022).

[31] Jin W, Li W, Khandekar C, Orenstein M, Fan S (2022). Laser cooling assisted thermal management of lightsails. ACS Photonics.

[32] Feng, D. et al. Temperature-dependent optical properties of materials for lightsail applications in Metamaterials, Metadevices, and Metasystems 2022 (2022), PC1219515.

[33] Manchester Z, Loeb A (2017). Stability of a light sail riding on a laser beam. Astrophys. J..

[34] Swartzlander GA (2017). Radiation pressure on a diffractive sailcraft. JOSA B.

[35] Ilic O, Went CM, Atwater HA (2018). Nanophotonic heterostructures for efficient propulsion and radiative cooling of relativistic light sails. Nano Lett..

[36] Atwater HA (2018). Materials challenges for the starshot lightsail. Nat. Mater..

[37] Chu Y-JL, Tabiryan NV, Swartzlander GA (2019). Experimental verification of a bigrating beam rider. Phys. Rev. Lett..

[38] Ilic O, Atwater HA (2019). Self-stabilizing photonic levitation and propulsion of nanostructured macroscopic objects. Nat. Photonics.

[39] Srivastava PR, Chu Y-JL, Swartzlander GA (2019). Stable diffractive beam rider. Opt. Lett..

[40] Siegel J (2019). Self-stabilizing laser sails based on optical metasurfaces. ACS Photonics.

[41] Salary MM, Mosallaei H (2020). Photonic metasurfaces as relativistic light sails for doppler-broadened stable beam-riding and radiative cooling. Laser Photonics Rev..

[42] Shirin A (2021). Modeling and Stability of a Laser Beam-Driven Sail.

[43] Holdman GR (2022). Thermal runaway of silicon-based laser sails. Adv. Opt. Mater..

[44] Gieseler N, Rahimzadegan A, Rockstuhl C (2021). Self-stabilizing curved metasurfaces as a sail for light-propelled spacecrafts. en. Opt. Express.

[45] Salary MM, Mosallaei H (2021). Inverse design of diffractive relativistic meta-sails via multi-objective optimization. Adv. Theory Simul..

[46] Kumar A, Kindem D, Ilic O (2021). Optomechanical self-stability of freestanding photonic metasurfaces. Phys. Rev. Appl..

[47] Gao R, Kelzenberg MD, Kim Y, Ilic O, Atwater HA (2022). Optical characterization of silicon nitride metagrating-based lightsails for self-stabilization. ACS Photonics.

[48] Brewer J (2022). Multiscale photonic emissivity engineering for relativistic lightsail thermal regulation. Nano Lett..

[49] Drobny J (2021). Damage to relativistic interstellar spacecraft by ISM impact gas accumulation. Astrophys. J..

[50] Timmons T, Bailet G, Beeley J, McInnes C (2021). Mars atmospheric characterization with a ChipSat swarm. J. Spacecr. Rockets.

[51] Duplay E, Bao ZF, Rosero SR, Sinha A, Higgins A (2022). Design of a rapid transit to Mars mission using laser-thermal propulsion. Acta Astronaut..

[52] Popova E, Efendiev M, Gabitov I (2017). On the stability of a space vehicle riding on an intense laser beam. Math. Methods Appl. Sci..

[53] Hughes GB (2014). Optical Modeling for a Laser Phased-array Directed Energy System.

[54] David Yaylali, Aerospace Engineering - Programming and Computation. https://www.asthecroworbits.com/computation.html (2022).

[55] Curtis HD (2020). Orbital Mechanics for Engineering Students.

[56] NASA. General Mission Analysis Tool (GMAT)–GSFC Open Source Software: https://opensource.gsfc.nasa.gov/projects/GMAT/index.php.

[57] Sweeting MN (2018). Modern small satellites-changing the economics of space. Proc. IEEE.

[58] Ridenoure RW (2016). Testing the lightsail program: Advancing solar sailing technology using a cubesat platform. J. Small Satell..

[59] Burkhardt, Z., Perakis, N. & Welch, C. Project Glowworm: Testing Laser Sail Propulsion in LEO in International Astronautical Congress 2018 (2018).

[60] Bishop, R. L., Clemmons, J. H., Barjatya, A. & Walterscheid, R. L. The Low- Latitude Ionosphere/Thermosphere Enhancements in Density (LLITED) Mission in (2017).

[61] Abulgasem S (2021). Antenna designs for cubesats: A review. IEEE Access.

[62] Davarian F (2020). Improving small satellite communications in deep space-A review of the existing systems and technologies with recommendations for improvement. Part I: Direct to earth links and smallsat telecommunications equipment. IEEE Aerosp. Electron. Syst. Mag..

[63] Clauss, R., Shell, J. & Reid, M. Low-Noise Systems in the Deep Space Network Deep space communications and navigation series - Jet Propulsion Laboratory and California Institute of Technology (2008).

[64] Wertz JR, Larson WJ, Kirkpatrick D, Klungle D (1999). Space Mission Analysis and Design.

[65] Liddle JD, Holt AP, Jason SJ, O’Donnell KA, Stevens EJ (2020). Space science with CubeSats and nanosatellites. Nat. Astron..

[66] Blue Canyon Technologies, https://www.bluecanyontech.com/components https://www.bluecanyontech.com/components (2022).

[67] Freeman A (2020). Exploring our solar system with CubeSats and SmallSats: The dawn of a new era. CEAS Space J..

[68] Klesh AT (2013). INSPIRE: Interplanetary NanoSpacecraft Pathfinder in Relevant Environment.

[69] Yost B (2021). State-of-the-Art Small Spacecraft Technology.

[70] Knap V, Vestergaard LK, Stroe D-I (2020). A review of battery technology in CubeSats and small satellite solutions. Energies.

[71] Trofimov SP, Ovchinnikov MY (2018). Performance scalability of square solar sails. J. Spacecr. Rockets.

[72] Kezerashvili RY (2009). Thickness requirement for solar sail foils. Acta Astronaut..

[73] Fernandez, J. M. Advanced deployable shell-based composite booms for small satellite structural applications including solar sails in International Symposium on Solar Sailing 2017 (2017).

[74] Taghavi M, Salary MM, Mosallaei H (2022). Multifunctional metasails for selfstabilized beam-riding and optical communication. Nanoscale Adv..

[75] Yu N, Capasso F (2014). Flat optics with designer metasurfaces. Nat. Mater..

[76] Evlyukhin AB, Matiushechkina M, Zenin VA, Heurs M, Chichkov BN (2020). Lightweight metasurface mirror of silicon nanospheres. Opt. Mater. Express.

[77] Jin W, Li W, Orenstein M, Fan S (2020). Inverse design of lightweight broadband reflector for relativistic lightsail propulsion. ACS Photonics.

[78] Heller R, Hippke M, Kervella P (2017). Optimized trajectories to the nearest stars using lightweight high-velocity photon sails. Astron. J..

[79] Perakis N, Hein AM (2016). Combining magnetic and electric sails for interstellar deceleration. Acta Astronaut..

[80] Creech, S., Guidi, J. & Elburn, D. Artemis: An Overview of NASA’s Activities to Return Humans to the Moon in IEEE Aerospace Conference (2022).

[81] Johnson L, Young R, Montgomery E, Alhorn D (2011). Status of solar sail technology within NASA. Adv. Space Res..

[82] Johnson L (2011). NanoSail-D: A solar sail demonstration mission. Acta Astronaut..

[83] Fu B, Sperber E, Eke F (2016). Solar sail technology-A state of the art review. Prog. Aerosp. Sci..

[84] Vatankhahghadim B, Damaren CJ (2021). Solar sail deployment dynamics. Adv. Space Res..

[85] Plainaki C (2016). Planetary space weather: Scientific aspects and future perspectives. J. Space Weather Space Clim..

[86] NASA’s Navigation and Ancillary Information Facility (NAIF). https://naif.jpl.nasa.gov/naif/toolkit.html (2022).

